# Exploring Potential Referral Pathways for Renal Artery Denervation and Developing a Centre of Excellence in Ireland

**DOI:** 10.1007/s42399-024-01647-9

**Published:** 2024-03-16

**Authors:** Niall Leahy, Max Wagener, Shirjeel Shahzad, Darragh Murphy, Amy McMorrow, Eileen Coen, Ruth Sharif, Faisal Sharif

**Affiliations:** 1 Cardiology Department, University Hospital Galway, Saolta Healthcare Group, Newcastle Road, Galway, H91 YR71 Ireland; 2 University of Galway, University Road, Galway, Ireland

**Keywords:** Renal denervation, Hypertension, Guidelines, Referral pathways, Centre of excellence

## Abstract

Arterial hypertension is one of the most significant and prevalent risk factors for cardiovascular disease. Despite widespread awareness of the condition, as well as a multitude of available antihypertensive drug classes, rates of uncontrolled hypertension remain high on a global scale. Frequently, poor compliance with anti-hypertensive medication plays a big role in patients’ inability to attain adequate blood pressure control. In individuals with resistant and/or uncontrolled hypertension, renal denervation is an emerging device-based therapy that has shown to be efficacious and safe in reducing blood pressure in several sham controlled trials. Additionally, it represents a treatment option for patients intolerant to oral pharmacotherapy.

University Hospital Galway has been performing renal denervation procedures over the past number of years within multicentre, international sham-controlled trials and registries. Representing a novel and emerging antihypertensive treatment option, sources of referral for renal denervation are diverse and multiple; thus, there is an unmet need for standardised referral structures in Ireland. Herein, we review current and developing referral pathways for renal denervation at our institution, and discuss streamlined patient management and requirements to establish a centre of excellence.

## Introduction

Arterial hypertension remains one of the most prevalent and modifiable risk factors in cardiovascular disease and a major driver for cardiovascular morbidity and mortality worldwide [1]. The high prevalence (over a billion people globally) and low control rates (18–23%) have a significant impact on healthcare systems and societies, through short- and long-term consequences which include myocardial infarction, chronic kidney disease, and stroke [1, 2]. Focusing on Ireland specifically, observational data has shown that the prevalence of hypertension in adults over the age of 50 is over 60%, and this figure will continue to grow as our national life expectancy continues to rise. Furthermore, amongst Irish adults on anti-hypertensive treatment, only approximately 50% have controlled blood pressure readings based on current guidelines [3–6]. A large-scale meta-analysis has proven the reduction in major cardiovascular events by optimising blood pressure (BP) control [7].

Whilst physicians have a variety of oral medication classes at their disposal, BP control rates remain suboptimal, owing largely due to partial or complete non-adherence to the medication [8]. In fact, up to 50% of individuals become partially or fully non-adherent within a year of commencing treatment [8]. ‘Resistant hypertension’ is defined by the European Society of Cardiology (ESC) as uncontrolled office blood pressure (BP) (systolic blood pressure (SBP) ≥ 140 mmHg and/or diastolic blood pressure (DBP) ≥ 90 mmHg), confirmed by ambulatory BP monitoring (ABPM) or home BP measurement, despite the implementation of appropriate lifestyle measures and use of three or more antihypertensive agents, including a diuretic [5, 9]. Diagnosis of resistant hypertension requires the exclusion of secondary hypertension. Furthermore, pseudo-resistant hypertension which is frequently due to medication non-compliance should be excluded [5].

To date, there have been several sham-controlled trials which have demonstrated the blood pressure lowering efficacy and safety of renal denervation (RDN) [10–16]. Considering the elevated cardiovascular risk in patients with uncontrolled and/or resistant arterial hypertension, RDN is proposed as a complimentary treatment option by the ESC Council on Hypertension and the European Association of Percutaneous Cardiovascular Interventions (EAPCI) [9]. Furthermore, in individuals unable to tolerate long-term antihypertensive agents or individuals who express a preference to undergo RDN, RDN may be considered following discussion with an expert in the clinical field in a tailored, shared decision-making process [9].

**Fig. 1:**
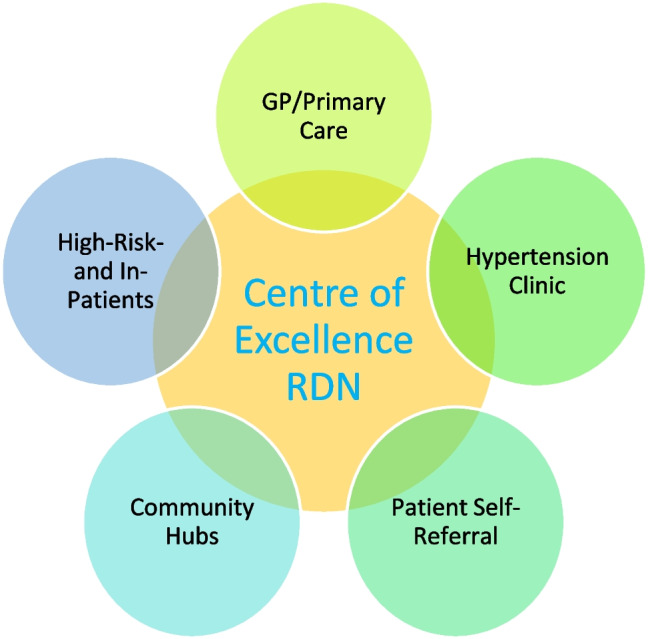
Sources of referral (*GP—general practitioner*,* RDN—renal denervation*)

University Hospital Galway is one of few centres in Ireland that have been performing renal denervation over the past number of years for patients within multicentre, international sham-controlled trials and registries. Herein, we review different referral pathways for RDN (Fig. 1), to streamline the management of patients with uncontrolled and/or resistant hypertension and optimise their outcomes.

### General Practitioner (GP)/Primary Care

Primary care is the central point of medical contact for the majority of patients in the community. The prevalence of hypertension amongst adults has increased from 650 million to over 1.28 billion over the last 30 years, and therefore hypertension is likely one of the most encountered comorbidities affecting GPs’ patients [1, 17]. ESC guidelines on the management of patients with arterial hypertension were published in 2018 and outline the recommended blood pressure targets and respective treatment options for attaining these targets [5]. Amongst patients encountered in primary care with uncontrolled hypertension, further investigations to exclude secondary hypertension and to detect resistant hypertension may be warranted. Furthermore, in patients with resistant hypertension, there is likely an underappreciation amongst GPs towards the role of RDN.

The majority of patients with hypertension are aware of some of the associated risks, such as stroke and myocardial infarction [18]. However, unfortunately, this does not guard against poor compliance with anti-hypertension medication [1, 19]. The psychological, financial, and physical burden, associated with often lifelong ingestion of oral medication, is probably significantly under-recognised and under-appreciated by clinicians. Surveys seeking to elucidate patients’ views on taking anti-hypertensive medication have found that up to 80% have reservations about taking them for one or more reasons [19]. Whilst advances in medical research and drug design have yielded exciting new developments in the world of cardiology-based pharmacological interventions, this enthusiasm must therefore be tempered by the realisation that our patients very often are simply unable, or unwilling, to take medications [5]. In the presence and absence of oral antihypertensive treatment, RDN has shown to provide sustained blood pressure reductions [10–15]. As specified in the ESC Consensus statement on the use of renal denervation in the management of hypertension, renal denervation is a possible treatment modality for patients unable to tolerate antihypertensive medications, or patients who express a preference to undergo the procedure, following a shared decision-making process [9]. GPs are therefore at a unique vantage point in terms of being aware as to which of their patients may be suitable for a RDN procedure. An efficient referral system for renal denervation would be highly beneficial to primary care physicians.

### Hypertension Clinic

In Galway, a multidisciplinary, consultant—led, hypertension clinic operates on a fortnightly basis in Merlin Park Hospital, which is affiliated with University Hospital Galway. It is especially tailored to young patients with uncontrolled hypertension, in whom there is a strong index of suspicion for secondary hypertension. The multidisciplinary nature of the clinic ensures that expert opinion from the specialties centrally associated with hypertension—Cardiology, Endocrinology, and Nephrology—is being provided. Standard operating procedures (SOPs) are in place to ensure that the requisite investigations to diagnose causes of secondary hypertension (Table 1) are performed in accordance with best practice guidelines. Furthermore, if true resistant and/or uncontrolled hypertension is confirmed, these specialised clinicians can evaluate a patient’s indication and suitability for RDN. Therefore, the hypertension clinic represents a key location where the procedure can be introduced and explained to the patient, and if they are agreeable, the patient can be referred to the centre of excellence.

**Table 1:** Common causes of secondary hypertension [20]

Common causes of secondary hypertension
Obstructive sleep apnoea
Renal parenchymal disease
Renal artery stenosis
Thyroid disease
Phaeochromocytoma
Cushing’s syndrome
Primary aldosteronism
Coarctation of the aorta
Unhealthy lifestyle-induced hypertension, e.g. C type hypertension (CtH)

### Patient Self-referral

Underpinning the inclusion of ‘patient self-referral’ as an important referral pathway is the significant shift which has occurred in recent years in overall patient attitude, awareness, and engagement in management of their specific health condition, as well as their involvement in ongoing research in that clinical field. In acknowledgement of this, the role of patient and public involvement (PPI) in clinical research has grown significantly over the last number of years, with evidence to show that PPI improves the quality and outcomes of research [21]. In recognition of this, the Health Research Board (HRB) in Ireland has an ongoing strategy and implementation plan to support PPI within the HRB and in associated projects and programmes [22]. Furthermore, patient-reported outcome measures (PROMs) are being increasingly incorporated into healthcare and clinical research given the valuable information that they can provide regarding the management of a particular health condition [23]. Fundamentally, PROMS provide insight into the effectiveness of healthcare from the patient’s perspective. This information is invaluable to help shape clinical practice and guide further research projects. Renal denervation is still a relatively new treatment modality for hypertension and thus there is continued research in this field. Involving patients and the public from the outset in this research is therefore of utmost importance.

Establishing a pathway for patients to self-refer for consideration for renal denervation recognises the important shift that has occurred in healthcare which empowers patients to be an active participant in the decision-making on management of their health condition.

### Community Hubs

The advent of SláinteCare has heralded the establishment of the Enhanced Community Care (ECC) pathway for Cardiology in the West of Ireland, which has led to the introduction of community based ‘hubs’ which aim to help shift the management of chronic cardiovascular conditions primarily to the community setting. Heart failure patients are an example of one cohort whose care is being redirected towards the community based setting, close to the individual’s geographical residency within the county. The management of these hubs is being overseen by two consultant cardiologists with the help of clinical nurse specialists and advanced nurse practitioners. Once these services are fully operational, it will be of enormous benefit to the management of patients with chronic cardiovascular conditions such as heart failure.

Given the likely high prevalence of hypertension in patients attending these clinics, the community hub setting is envisaged to play a key role in the referral pathway for renal denervation.

### Inpatient Hospital Setting

The high turnover of patients admitted to University Hospital Galway under the Cardiology and General Medicine services provides teams with an ideal opportunity to identify patients who have uncontrolled hypertension. Patients who are admitted for multiple days will have blood pressure readings frequently throughout any given day, and whilst this does not provide clinicians with as accurate of an indication of average blood pressure compared to that which is derived from ambulatory measurement, persistently elevated readings will signal that the patient has uncontrolled hypertension. Additionally, these readings are taken in the setting of patients being witnessed taking their anti-hypertensive medications; thus, issues regarding compliance are negated and the veracity of the blood pressure readings are possibly greater. In many cases, patients may already be on multiple antihypertensive agents and are therefore likely meeting the criteria for resistant hypertension. Clinicians should be alert to these patients and once identified, having a structured pathway in place will allow these patients to be referred for consideration for renal denervation. This pathway will involve patients being seen by a dedicated renal denervation CNS whilst an inpatient, and if deemed to be possible candidates for renal denervation, they should be referred for further consideration and to outrule secondary causes of hypertension, whilst having a 24-h blood pressure monitor performed by their GP in the interim.

**Fig. 2:**
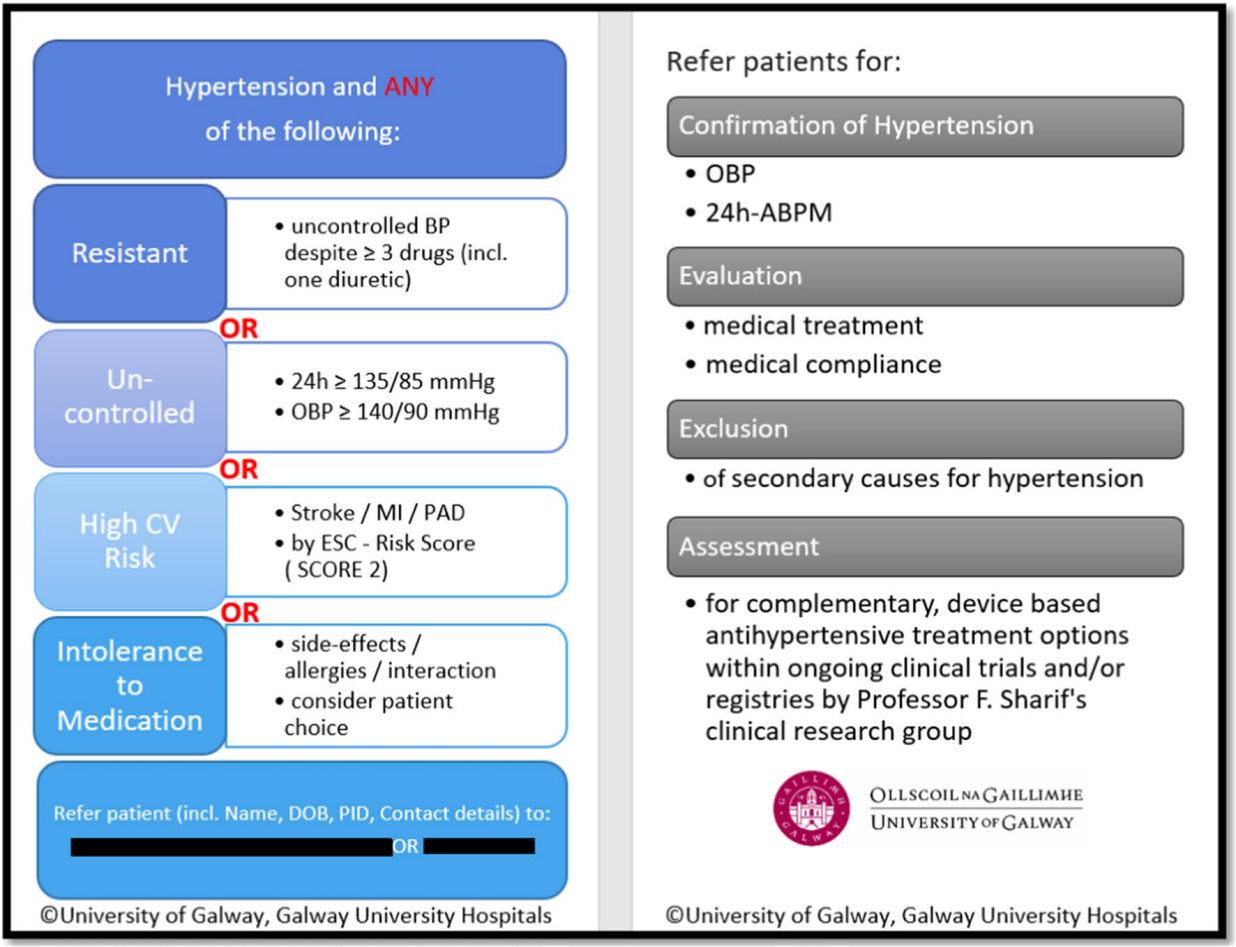
Referral card for inpatient screening for resistant and uncontrolled hypertension. (*ABPM—ambulatory blood pressure monitor*,* BP—blood pressure*,* CV—cardiovascular*,* DOB—date of birth*,* OBP—office blood pressure*,* PID—patient identification number*)

To create awareness and a screening aid, referral cards (Fig. 2) have been designed by the Renal Denervation research team in University Hospital Galway. These pocket-size cards, outlining the diagnostic components of uncontrolled and/or resistant hypertension, are currently in circulation within the hospital for inpatient teams.

## Discussion

### Logistical Considerations—Cardiac Catheterisation Laboratory Availability

The ESC Council on Hypertension & EAPCI consensus statement on renal denervation in the management of hypertension specifies that centres performing the procedure should have experts on hypertension and percutaneous cardiovascular interventions, which University Hospital Galway has. As well as this, centres should have a catheterisation laboratory, coronary unit, and access to emergency vascular surgery facilities [9]. In our opinion, centres performing RDN should assess patient and peri-procedural data in auditable databases for quality control and research purposes.

There are currently two cardiac catheterisation laboratories which are operational in University Hospital Galway performing a range of elective diagnostic and interventional procedures, as well as emergency procedures on a 24/7 basis.

Allocation of time to perform elective renal denervation on a regular basis in addition to the services already offered requires solid evidence for unmet need in this area. Whilst the prevalence of true resistant hypertension is difficult to state since many people with uncontrolled hypertension have poor adherence or suboptimal medication regimens, studies have nevertheless indicated that it is indeed sizeable. A systematic review and meta-analysis involving over 3.2 million people with hypertension on antihypertensive agents published in 2019 found that the global prevalence of true resistant hypertension was 10.3%, with this higher in elderly patients and those with chronic kidney disease [24]. With the average life expectancy in Ireland now one of the highest in Europe, we can intuitively expect this figure to be slightly higher today [4]. Therefore, there are a considerable proportion of individuals who may benefit from renal denervation to help attain target blood pressure control and thus optimise their cardiovascular health from both a primary and secondary prevention perspective.

### Destination for Referrals—Renal Denervation Centre of Excellence

Central to optimising the efficiency of any referral pathway is a clear destination designed to facilitate patient management. At University Hospital Galway, referrals for screening and the procedure are sent to our Renal Denervation Centre of Excellence, which is integrated with and coordinated by the cardiovascular research group under the stewardship of a Consultant Interventional Cardiologist. After thorough assessment and confirmed diagnosis of true resistant and/or uncontrolled hypertension, an open and detailed discussion with the patient regarding indication, benefits, and potential risks of renal denervation can be carried out. Prior to this discussion, secondary causes of hypertension should be outruled if not already done, and efforts to optimise patients’ lifestyle should be made given that this may be contributing to their hypertension [25]. Following this shared decision-making process, the procedure can be planned in cooperation with the cath lab coordinators.

### Follow-up

Following a renal denervation procedure, patients should be monitored in a Coronary Care Unit for access site complications such as haematoma and pseudoaneurysms. Fortunately, there are no specific safety concerns associated with renal denervation beyond the expected complication rates of transfemoral access which occur in less than 1% of cases [9–15]. Therefore, patients can expect a prompt discharge from hospital post-procedure.

At our centre, patients undergo standardised follow-ups within the local registry with 24-h ABPM at 3 and 6 months. Patients have the contacts of our onsite nurse in case of complications and questions regarding the procedure, as well as the antihypertensive medical treatment. Patients should be counselled that further meaningful reductions in blood pressure can be seen up to 36 months post-procedure [9]. Patients should be encouraged to continue taking their oral antihypertensive medication, and aspirin should be taken for at least 1 month post-procedure. A 24-h ambulatory blood pressure check with the patient’s GP should be performed, and in selected cases, ultrasound imaging at 6 months and 1 year may be needed to exclude renal artery stenosis [26].

Long-term patient outcomes will be important to assess going forward as higher volumes of the procedure are performed. A recent pilot project, renal denervation focus group, involving patients who underwent renal denervation in Galway University Hospital, aimed to assess patient awareness of arterial hypertension and its consequences, as well as assess patients’ views on peri- and post-procedural aspects and their associated quality of life. Most patients reported a subjective benefit after treatment and no significant side effects other than mild bruising were reported. No negative impact of renal denervation on quality of life was reported.

## Conclusion

This review details the proposed referral pathways for a structured and efficient renal denervation programme at a tertiary university hospital. As outlined above, the sources of referrals are diverse and target a large patient cohort, with the ultimate destination for the referrals being the Renal Denervation Centre of Excellence.

Despite a significant development in the armamentarium of pharmacological interventions for hypertension over the past few decades, there remains a significant proportion of individuals with uncontrolled hypertension and thus at high risk of future primary and secondary cardiovascular events. Device-based therapy offers an exciting complementary method of treatment with positive effects on blood pressure demonstrated in sham-controlled trials. With a focused blueprint for a referral pathway in place at University Hospital Galway, we envisage that this centre will continue to grow and develop into one of the leading centres in the country in performing renal denervation. A limitation of our review is that referral pathways are bound to local healthcare structures and circumstances. Thus, the above mentioned may not apply to every upcoming centre of excellence for RDN and may need adaptations to be practicable under local circumstances.

## Data Availability

Proposing a change in practice, no patient related data and/or material was used for this manuscript.
